# Post-Migration Stressors and Health-Related Quality of Life in Refugees from Syria Resettled in Sweden

**DOI:** 10.3390/ijerph19052509

**Published:** 2022-02-22

**Authors:** Mathilde Sengoelge, Alexander Nissen, Øivind Solberg

**Affiliations:** 1 Department of Health Sciences, The Swedish Red Cross University College, 121 41 Huddinge, Sweden; 2 Department of Global Public Health, Karolinska Institutet, 171 77 Stockholm, Sweden; a.f.w.nissen@nkvts.no (A.N.); oivind.fjeld-solberg@nca.no (Ø.S.); 3 Division for Forced Migration and Refugee Health, Norwegian Centre for Violence and Traumatic Stress Studies, 0484 Oslo, Norway; 4 Norwegian Church Aid, 0130 Oslo, Norway

**Keywords:** preference-based health-related quality of life, refugee, EQ–5D–5L, post-migration stressors

## Abstract

The link between post-migration stressors and mental ill health is well documented in refugees resettled in high-income host countries, but the consequences of these stressors on refugees’ health-related quality of life (HRQoL) are less known. This study examined the association between post-migration stressors and HRQoL among Syrian adult refugees resettled in Sweden using a preference-based value set obtained from the general Swedish population. A total of 1215 Syrian adults, ages 18–64 years, granted residency in Sweden, responded to a postal questionnaire in 2016 regarding various aspects of their resettlement. The European Quality of Life Five Dimensions Five Level (EQ–5D–5L) questionnaire was used to assess HRQoL through an EQ–5D–5L index score (range; 0=dead to 1=full health). The index score was preference weighted using a Swedish population value set. Predictors were four self-reported post-migration stressors related to daily living in the host country: financial strain, social strain, competency strain and perceived discrimination divided into low, medium and high levels of experienced stress. Multivariable linear regression models were employed to assess the association between post-migration stressors and HRQoL index score, adjusting for potentially traumatic events in the pre- and peri-migration phase as well as sociodemographic confounders/covariates (sex, age, education, civil status, immigration year). The Syrian refugees had a mean EQ–5D–5L index score of 0.863 (SD = 0.145). There was strong evidence of a negative dose-response association in both unadjusted and adjusted models between HRQoL and the post-migration stressors financial strain and social strain—i.e., there was a stepwise, and statistically significant, decrease in HRQoL when going from low to medium to high strain. Competency strain and discrimination were only associated with lower HRQoL when experienced at high levels in fully adjusted models. High exposure to potentially traumatic experiences before or during flight was also associated with lower HRQoL. Syrian refugees resettled in Sweden reported a lower HRQoL than the general Swedish population and lower than age-matched Swedish adults. The present study results point to the possible adverse effects of post-migration stressors on HRQoL.

## 1. Introduction

The link between stressors pre-, peri- and post-migration and mental ill health in refugees resettled in high-income host countries is well documented [1,2,3,4], and a growing number of studies are exploring how these stressors are associated with the broader concept of quality of life (QoL). A review of refugee populations in high-income countries concluded that social networks and social integration was positively associated with QoL, whereas mental ill health (e.g., depression or PTSD) was negatively associated with QoL [5]. When comparing QoL in general and clinical populations of refugees in community settings, a recent review found that there was a wide heterogeneity in the scores, and domain-specific patterns of QoL varied across the two groups [6]. Both reviews acknowledge the complexity and diversity of the refugee experiences and the variety of elements that may affect QoL.

Indeed, QoL is a complex, multidimensional concept developed to encompass an individual’s perception of one’s physical and psychological state, level of independence, social relationships, personal beliefs and relationship to the environment [7,8]. Moreover, health is a major component of QoL, yet the related term health-related quality of life (HRQoL) has been inconsistently defined and operationalized in the scientific literature of migrants [9]. For example, use of the term HRQoL when referring to a measure of self-perceived health status, or when stating that research is focusing on HRQoL when the instrument measures “overall” QoL [10]. The key to HRQoL research is an individual’s *evaluation of* one’s health state, as compared to solely reporting on it [11,12]. The term HRQoL should be utilized when either: (a) exploring how health affects QoL using separate measures of each and statistically exploring how they relate to one another, or (b) examining the utility associated with different health states using health status questionnaires with an attached value set [13].

Preference-based HRQoL studies both assess and apply preference weights for different health states. This valuation is summarized as a health index score anchored on a 0 (dead) to 1 (perfect health) scale [14]. Such measures have been increasingly used to determine whether there has been a change in HRQoL in the population [15]. Examples of such measures include the SF−6D [16] and the European Quality of Life Five Dimensions known as the EQ–5D [17]. Few studies in the published literature to date have explored HRQoL in refugee populations using such a preference-based approach. One study of 133 Syrian refugees in Germany found that the HRQoL of the refugees reported was lower compared to a representative German population sample [18]. A prior publication by our research group used the EQ–5D–5L index score to examine gender differences in HRQoL in Syrian adult refugees resettled in Sweden but based on the United Kingdom population data as no value set was available for the Swedish population at the time [19]. The study found that male sex, younger age, living with a partner and social support were positively associated with HRQoL [19].

Moreover, potential determinants of HRQoL in refugee populations might be related to the level of trauma experienced before and during flight [20], including exposure to torture [21]. Other determinants may involve stressors experienced in the resettlement phase [22]. These post-migration stressors can be family-related (e.g., family separation, conflicts with family), related to poor social integration and weak social networks (e.g., no or low number of friends from within or outside one’s ethnic community, low participation in activities), or related to financial or housing difficulties, poor host language proficiency and/or unemployment [23]. All of these resettlement stressors have been shown to be associated with higher levels of negative mental health outcomes, particularly post-traumatic stress disorder, anxiety and depression [3,24]. HRQoL may also likely be linked to cultural values in the perception of one’s own state of health [25].

In sum, there is a scarcity of studies that utilize the full potential of HRQoL measures in refugee populations examining preference-based HRQoL and in relation to flight-related trauma exposure and post-migration stressors. Host countries need to obtain an understanding of the HRQoL of its refugee population in order to allocate resources and monitor the effectiveness of broad community interventions. With this knowledge, host countries can more effectively identify and support positive resettlement interventions, services and policies that mitigate unmet needs. Thus, the aim of the present study was to establish a benchmark reporting of HRQoL among a sample of Syrian adult refugees resettled in Sweden using preference-weighted data, and to explore how post-migration factors influence HRQoL.

## 2. Materials and Methods

### 2.1. Study Population

Eligible participants for the study included all adult refugees from Syria who were given permanent residency in Sweden and resettled in the country between 2011 and 2013. A random sample of 4000 refugees was drawn from a sampling frame of *N* = 9662 identified through the Swedish Population Registry. All sampled refugees were invited to participate in a postal questionnaire survey in 2016 on self-reported health, pre- and peri-migration experiences and various aspects of their resettlement, drawn up in Swedish and back-translated into Arabic. Of the invited refugees, 1215 returned the questionnaire (response rate = 30%). More detailed information on design, sampling and recruitment strategies and potential study population bias has been previously published [26].

### 2.2. Measures

#### 2.2.1. European Quality of Life Five Dimensions Five Level (EQ–5D–5L)

The EQ–5D–5L is a well-known indirect, generic measure of HRQoL consisting of five domains: mobility, self-care, usual activities, pain/discomfort and anxiety/depression [27]. For each domain, participants endorse one of five levels of functioning ranging from no problems (=1), slight problems (=2), moderate problems (=3), severe problems (=4) to unable to/extreme problems (=5). A full, 5-digit scoring profile, or health state, is created by putting each domain score after one another in the order outlined above (starting with mobility and ending with anxiety/depression). For example, someone reporting ‘no problems’ on all five domains has a profile of 11,111, whereas someone with ‘extreme problems’ in the domains usual activities and anxiety/depression and ‘no problems’ in the other three domains has a profile of 11515. In total, there are 3125 possible combinations of domains scores and thus 3125 possible health states. In order to compare the perceived relative value or utility of different states, preference data are obtained from people experiencing these states by asking their willingness to trade time in their current state for time in ‘perfect health’ (time trade-off method), and this preference is converted to a number between 0 and 1. People who are perfectly happy with their health will not be willing to trade off any time and they define the ‘perfect health’-end of the spectrum and are given an index score of 1 (10/10 = 1). People who struggle with severe health issues may be willing to trade, say, 10 years in their current health for 1 year in perfect health (hypothetically), and are given an index score of 0.1 (1/10 = 0.1). The closer the index score is to 1, the better the associated health and HRQoL. A value set for a population is a complete coding scheme where each of the possible 3125 health states have an assigned index score based on preference data from the population in question. Using the example above, the profile 11111 will have an index score of 1.0 (perfect health), whereas the profile 11515 may have an index score of, say, 0.70 (this depends on how the people with this profile in the population responded to the time trade-off question). The present study uses a value set from the Swedish general population to estimate HRQoL index scores for respondents [28]. 

#### 2.2.2. Post-Migration Factors

The Refugee Post-Migration Stress Scale was used to measure four domains of post-migration strain related to resettlement in the host country: (1) financial strain; (2) social strain; (3) competency strain; (4) perceived discrimination [29]. Financial strain relates to material and economic hardship that could affect integrity, independence, dignity and well-being (example: ‘Worry about unstable financial situation’). Social strain relates to social hardship, e.g., feeling isolated or frustrated due to loss of status (example: ‘Feeling excluded or isolated in the Swedish society’). Competency strain relates to feelings of inadequacy of host-country specific skills needed to successfully navigate and function in daily life (example: ‘Bothering difficulties communicating in Swedish’). Perceived discrimination asks about experiences of unfair treatment in Sweden, either verbally or nonverbally, on the basis of prejudice (example: ‘Feeling disrespected due to my national background’). All domains are comprised of three items, except perceived discrimination that has four items. Respondents were asked to indicate how frequently they experience each item on a scale ranging from 1 = never to 5 = very often. Please refer to Figure S1 for the distribution of responses on individual items. Respondents were categorized into low, medium and high strain for each of the four domains. The low-strain group had a maximum score of 2 = seldom for all items within a given domain. The high-strain group answered 4 = often or 5 = very often on all items within a given domain, and the medium-strain group consisted of the remaining. Cronbach’s alpha for the four domains ranged between 0.80 and 0.84.

#### 2.2.3. Sociodemographic and Pre-Migration Trauma Exposure

Potential confounders included were sociodemographic variables, e.g., sex, age, education, civil status and year of immigration to Sweden. Potentially traumatic experiences (PTEs) related to before (pre-flight) or during (peri-flight) migration were measured with the Refugee Trauma History Checklist, tested and validated in a sample of Syrian asylum seekers in Sweden [30]. The scale asks about eight PTEs prior to flight, and the same eight during flight, for a total of 16 PTE items (e.g., ‘War at close quarter’ and ‘Forced separation from family or close friends’). A PTE adversity ratio (PTE-AR) introduced by Steel et al., was calculated as the number of endorsed PTEs divided by the total number of PTEs inquired about and categorized into: <0.2; 0.2–0.29; 0.3–0.39; ≥0.4 [2].

### 2.3. Statistical Analyses

Data were inspected for errors, outliers and missing values. Frequency distributions, simple summary statistics and cross tabulations were used to make the descriptive table. Sociodemographic variables were modelled as in prior studies by our group to facilitate comparison. Unadjusted and adjusted logistic regression were used to explore the association between post-migration stressors and the five domains in the EQ5 scale. Each domain was analyzed separately and answer choices were dichotomized with choice 1 (i.e., ‘no problem’) as the reference category and choices 2–5 (i.e., ‘slight problems’ or higher) defined as a case. In the adjusted analysis, missing was handled through listwise deletion with the total number contributing data to full models indicated in the relevant table. Odds ratios (ORs) with 95% confidence interval (95% CI) and associated *p*-values are presented.

Unadjusted and adjusted linear regression was used to investigate the association between post-migration stressors and HRQoL, with results reported through unstandardized regression coefficients with 95% CI and associated *p*-values. Standard regression coefficients were also estimated to make it easier to compare regression coefficients for different post-migration stressors. The standardized coefficients are only commented on in the text in order not to clutter tables. Missing values were handled through listwise deletion as in logistic regression (total number included in models is denoted in the table). Linear regression was deemed appropriate even if the outcome was skewed due to the large sample size (central limit theorem).

## 3. Results

Descriptive statistics of all respondents are shown in Table 1, as well as the proportion of respondents reporting at least ‘slight’ problems within each of the five dimensions of the EQ–5D–5L. Dimensions four (pain/discomfort) and five (anxiety/depression) were the two dimensions with the highest number of respondents reporting problems (over half in each dimension). Respondents with higher levels of post-migration stress reported problems in each of the five dimensions more frequently and had lower index scores. This occurred for all post-migration stressors. Furthermore, there was a clear dose-response pattern for each stressor. The high stress group reported more problems and had a lower mean index score than the medium stress group, which had more problems/a lower index score than the low stress group.

Table 2 presents unadjusted and adjusted logistic models of each dimension of the EQ–5D–5L regressed on post-migration stressors, controlling for pre- and peri-migration stress and sociodemographic variables. There was strong and consistent evidence in both the unadjusted and partially adjusted models that all post-migration stressors were associated with increased odds of reporting problems in all five dimensions of the EQ–5D–5L, with the exception of discrimination. In general, there were small to moderate attenuations of the ORs after controlling for sociodemographic variables, indicating that the confounding by these variables was not substantial. In the fully adjusted models with all post-migration stressors included together with pre- and peri-migration stress, the evidence for associations changed notably. Self-care (dimension 2) was no longer associated with any of the post-migration stressors, and the associations between competency strain and the EQ–5D–5L dimensions were markedly attenuated or no longer significant. A similar attenuation pattern was seen for social strain, though the association between social strain and dimension 4 (pain/discomfort) and 5 (anxiety/depression) remained highly significant. The strongest evidence was found for financial strain, which was strongly associated with all EQ–5D–5L dimensions except for self-care, with the odds of experiencing problems three- to fourfold higher in the high financial strain group compared to the low strain group (all *p*-values < 0.001).

In Table 3, unadjusted and adjusted linear regression models of the EQ–5D–5L index score are presented. There was strong evidence in both unadjusted and partly adjusted models that all four post-migration stressors were associated with a lower index score and there were clear dose-response patterns. When comparing standardized regression coefficients in partly adjusted models, financial strain and social strain were the two stressors associated with the greatest change in the standardized index score. In the fully adjusted model, there was very strong evidence that respondents reporting the highest level of financial and social strain had lower index scores compared to the lowest strain groups, with the standardized regression coefficient for financial strain much larger than that for social strain (*β* = −0.296 vs. *β* = −0.166, respectively). There was also evidence that respondents in the medium strain group for these two stressors had lower index scores compared to the low strain group, though the evidence was weaker. Respondents reporting high levels of competency strain and discrimination had lower index scores compared to their respective reference categories, though the standardized regression coefficients were notably smaller than for financial strain (*β* = competency strain = −0.108; *β* = discrimination = −0.094) and we found no evidence for an association for the medium strain group. Lastly, high exposure to potentially traumatic experiences before or during flight was associated with a lower HRQoL index score at the *p* < 0.01 level. 

## 4. Discussion

In the present study, refugees from Syria resettled in Sweden reported a lower HRQoL than an age-matched Swedish reference population [31] and lower than the general Swedish norm data from which the value set was derived [28]. A markedly higher proportion of refugees reported problems in the Anxiety/depression domain (62% vs. 37% in the Swedish norm data). In contrast, the proportion with reported problems in the domains Mobility, Self-care and Usual activities were comparable in both groups, whereas a somewhat lower proportion of refugees reported problems in the domain Pain/discomfort (55% vs. 68% in the Swedish norm data). Mental health is therefore the likely driver of the lower HRQoL reported by the refugee group. However, comparability of HRQoL index scores in the current study with those of the representative Swedish population samples is limited due to the younger age of the current sample. Approximately half of the refugees were under 40 years of age compared to 9% in the Swedish sample, and there exists a known and very strong negative association between age and HRQoL. The estimated mean index score is also somewhat higher than the 0.82 value found in a study of Syrian refugees resettled in Germany [18]. Possible explanations for this difference include the use of value sets obtained from different populations (Sweden versus Germany). Additionally, an inclusion criterion in the German study was the presence of mild to moderate symptoms of post-traumatic stress, whereas the present study used a general refugee population. Moreover, the German sample had a notably higher proportion of singles/divorced and civil status was strongly associated with HRQoL in our study.

To our knowledge, the present study is one of the first studies to estimate HRQoL using preference-weighted data in a large sample of refugees while exploring the association between HRQoL and post-migration stressors. Refugees who reported experiencing high levels of post-migration stressors had the lowest HRQoL scores. The stressors driving this association were firstly financial strain, followed by social strain.

The measure of financial strain used in the present study was related to material and economic hardship threatening integrity, independence, dignity and well-being. Refugees in Sweden receive financial assistance to cover basic needs for clothing and food, but the findings from this study suggest that many refugees nonetheless experience financial strain that may adversely and significantly impact HRQoL. This is supported by a study on Iraqi asylum seekers in the Netherlands, which found that socioeconomic living conditions was a more important predictor of reduced overall QoL than psychopathology [32]. While relatively few studies have explored the association between financial strain and QoL in refugee populations, there is solid evidence for the adverse association between financial strain and mental distress (for an overview see [4,24]), including a recent, longitudinal study from Australia providing consistent evidence across all time-points that economic stressors were positively associated with mental illness [1]. Limited access to employment and economic opportunities is key to explaining the economic hardship often faced by refugees. Employment rates of refugees resettled in high-income countries are typically under 20% in the first two years after arrival and then increase depending on the host country. However, refugees have been shown to continue having lower employment rates than other immigrants and natives, even ten years after migration [33]. Moreover, adequacy of income and employment have been shown to be significant predictors of general mental health for resettled refugees [34,35] and to moderate the adverse mental health effects of pre- and peri-migratory stressors experienced by refugees [36]. Barriers to employment identified by refugees include adverse effects of PTSD, problems with professional recertification and economic barriers to pursuing education [37].

In the case of social strain related to feelings of isolation and loss of status in the host country, a fair number of studies have found positive associations between social integration/support and QoL both in selected [38,39] and more general refugee populations [40,41,42]. Studies have further shown that social connectedness and support are key enablers for integration, health and well-being, with some evidence suggesting patterns are gender specific [43,44]. A recent, large German survey of 4325 resettled refugees found that contact with members of the host society, better host country language skills and being employed were related to reduced distress and higher levels of life satisfaction [45]. The negative relationship between social integration and mental distress has been documented in several studies [39,46,47], including in the abovementioned longitudinal study of refugees resettled in Australia [1]. The study found that loneliness during resettlement was positively associated with PTSD and severe mental illness over time, though the strength of the association fluctuated across timepoints, suggesting time-varying effects. A challenge when comparing findings from the present study to the existing literature is that the concept of social strain is defined and measured in different ways across studies. Whereas many studies focus primarily on loneliness and the lack of social networks, the present study also includes status loss and frustration at not being able to use skills and competence. In summary, the overall findings in the current study on financial and social strain are consistent with much of the available evidence to date linking both stressors to lower levels of positive mental health outcomes, such as QoL and well-being, and elevated levels of mental distress. 

Contrasting this overall picture is a recent systematic review by Hou and colleagues [48]. The review investigated both general distress and well-being/QoL in relation to post-migration everyday life stressors stratified into *subjective* (perceived emotional distress associated with different daily experiences), *interpersonal* (e.g., conflict, discrimination, isolation, lack of emotional support) and *material* (e.g., housing/neighborhood contexts, accommodation difficulties, employment-related issues, access to social or mental health services). Results showed consistent positive associations of daily stressors with general distress but, somewhat surprisingly, non-significant effect sizes between daily stressors and general well-being/QoL across seven studies. The authors concluded that more research focusing on domain-specific QoL in relation to stressors is needed. By using a single index value as the outcome measure for HRQoL, the present study took a somewhat opposite approach. The strong associations found between both financial and social strain and the single index HRQoL score suggest this approach may be reasonable. A large sample size increasing power may partly explain why significant associations were found in the present study and not in the studies included in the review by Hou [48]. Another possible explanation is that the present study used a preference-based approach to HRQoL. Our findings demonstrate that worse HRQoL among Syrian adult refugees living in Sweden may not be limited to mental health problems, but also extend to other dimensions of general health that in turn influence quality of life. This is important from a health determinants viewpoint as interventions to address this are limited by the complexity of resettlement hardships faced by this group as they attempt to acculturate into the host society. Future studies are needed to analyze the HRQoL of refugees in Sweden longitudinally, as compared to the general Swedish population and in refugees of different countries of origin. More research is also required to identify mechanisms that strengthen HRQoL, such as support networks, employment and education opportunities.

### Strengths and Limitations

Strengths of the study include random sampling from population-based registries, a large sample size and the use of well-established measures of key variables. A particular strength is that HRQoL was estimated by combining refugees’ HRQoL self-reported data with an experienced, preference-based value set obtained from the general Swedish population. The study thus follows expert advice that studies on HRQoL should move beyond looking solely at self-perceived health and/or general QoL, and instead focus on the *utility* associated with health, accomplished by using health status data in combination with an attached value set [13]. Our results represent a benchmark that can be used to evaluate changes in HRQoL in this sample over time or after participation in specific interventions or policies. Furthermore, information about the sociodemographic variables, except civil status, was retrieved from national, high-quality registers, reducing the risk of information bias.

A limitation is that self-reported health is likely linked to cultural values [25], thus, applying preference data from one culture to another, in this case adults in Sweden to adults with a refugee background from Syria living in Sweden, could lead to an index score with low validity. However, in the absence of available preference data from any refugee population, this may still be the best alternative. Another limitation is that the data were self-reported through a postal survey. Refugees with low HRQoL may have not been able to participate in the survey (non-responders), resulting in potential selection bias and a sample that is healthier than the target population.

## 5. Conclusions

Syrian refugees resettled in Sweden report a lower HRQoL than the general Swedish population and lower than age-matched Swedish adults. Findings of the present study also point to the adverse effects of post-migration stressors on HRQoL. Effective, community-based interventions are needed to reduce the financial and social strain experienced by this population. Moreover, multidimensional interventions are recommended to improve HRQoL in combination with advocacy and support to improve financial status and social competency skills. 

## Figures and Tables

**Table 1:** Descriptive characteristics of Syrian adult refugees resettled in Sweden.

Characteristic	Level	All Respondents		EQ–5D–5L—Percentage with Problems					ED5 Index Score	
		*n*	%	D1	D2	D3	D4	D5	Mean	(SD)
		1215	100.0	27.1	6.5	29.5	54.8	61.7	0.863	0.145
Gender	Male	763	62.8	23.8	5.7	27.4	50.3	60.5	0.871	(0.139)
	Female	452	37.2	32.4	7.9	32.8	62.2	63.8	0.851	(0.153)
Age	18–29	283	23.3	11.8	2.5	18.1	40.6	56.9	0.895	(0.119)
	30–39	400	32.9	20.7	4.7	23.9	49.2	59.9	0.878	(0.136)
	40–49	295	24.3	30.1	5.6	29.3	60.3	62.0	0.867	(0.138)
	≥50	237	19.5	53.1	15.9	52.8	74.6	70.4	0.792	(0.173)
Education	0–9 yrs	453	38.4	33.5	8.0	32.1	52.5	56.8	0.850	(0.160)
	10–12 yrs	255	21.6	23.5	5.7	27.8	52.4	62.6	0.872	(0.138)
	13–14 yrs	234	19.9	26.2	7.1	27.6	55.8	65.0	0.866	(0.138)
	≥15 yrs	237	20.1	20.3	5.1	27.8	61.6	67.4	0.872	(0.128)
Civil status	Married	771	63.4	30.4	6.6	31.7	57.3	60.0	0.864	(0.141)
	Unmarried	386	31.8	18.5	5.1	23.6	47.3	63.7	0.871	(0.147)
	Div./wid.	58	4.8	40.0	17.0	39.3	69.6	72.7	0.799	(0.174)
Year immigration	2008–2011	76	6.3	28.8	4.2	34.7	57.5	55.4	0.860	(0.151)
	2012	334	27.5	25.6	6.9	28.7	54.6	58.1	0.856	(0.158)
	2013	802	66.2	27.4	6.6	29.1	54.5	63.9	0.867	(0.139)
PTE adversity ratio	<0.20	279	24.4	17.3	5.1	21.9	45.3	49.6	0.899	(0.126)
	0.20–0 29	128	11.2	26.8	4.0	25.0	55.2	56.3	0.887	(0.113)
	0.30–0 39	250	21.9	26.1	6.6	30.9	54.4	61.6	0.865	(0.137)
	≥0.40	486	42.5	32.5	8.0	34.9	60.2	69.3	0.833	(0.162)
Financial strain	Low	284	23.8	10.4	1.4	10.4	30.1	34.8	0.937	(0.077)
	Medium	692	58.1	28.0	6.7	30.3	57.5	65.9	0.868	(0.130)
	High	216	18.1	45.0	11.8	50.7	77.0	83.5	0.759	(0.180)
Social strain	Low	326	27.8	15.6	2.2	13.8	33.7	37.7	0.926	(0.088)
	Medium	713	60.8	29.4	7.3	33.0	59.3	68.1	0.858	(0.140)
	High	133	11.4	41.5	11.6	46.9	81.1	86.4	0.748	(0.190)
Competency strain	Low	286	24.2	11.1	2.9	12.4	37.1	49.8	0.912	(0.104)
	Medium	787	66.6	28.3	6.0	32.4	57.9	63.1	0.861	(0.142)
	High	108	9.2	52.0	16.2	50.0	73.1	77.4	0.769	(0.186)
Discrimination	Low	748	63.4	27.5	6.9	27.7	51.7	55.6	0.873	(0.142)
	Medium	425	36.0	25.1	5.6	31.8	59.4	71.5	0.852	(0.142)
	High	7	0.6	57.1	28.6	42.9	85.7	85.7	0.601	(0.204)

D1 = Mobility; D2 = Self-care; D3 = Usual activities; D4 = Pain/discomfort; D5 = Anxiety/depression; PTE-AR: potentially traumatic experiences adversity ratio.

**Table 2:** Unadjusted and adjusted logistic regression models of having problems in domain-specific EQ–5D–5L.

Regression Models	Level	Mobility		Self-Care		Usual Activities		Pain/Discomfort		Anxiety/Depression	
		OR	95% CI	OR	95% CI	OR	95% CI	OR	95% CI	OR	95% CI
**Model 1—unadjusted †**											
Financial strain	Med	**3.34**	**(2.19–5.08)**	**4.94**	**(1.76–13.9)**	**3.77**	**(2.48–5.72)**	**3.14**	**(2.33–4.22)**	**3.63**	**(2.71–4.87)**
	High	**7.03**	**(4.39–11.3)**	**9.21**	**(3.15–26.8)**	**8.90**	**(5.57–14.2)**	**7.77**	**(5.16–11.7)**	**9.49**	**(6.12–14.7)**
Social strain	Med	**2.26**	**(1.60–3.19)**	**3.48**	**(1.56–7.75)**	**3.07**	**(2.15–4.38)**	**2.87**	**(2.17–3.79)**	**3.54**	**(2.68–4.68)**
	High	**3.86**	**(2.43–6.13)**	**5.77**	**(2.29–14.5)**	**5.53**	**(3.46–8.83)**	**8.44**	**(5.15–13.8)**	**10.5**	**(6.07–18.1)**
Competency strain	Med	**3.18**	**(2.12–4.76)**	**2.18**	**(1.01–4.67)**	**3.39**	**(2.30–4.98)**	**2.33**	**(1.76–3.08)**	**1.72**	**(1.31–2.27)**
	High	**8.69**	**(5.07–14.9)**	**6.57**	**(2.74–15.7)**	**7.06**	**(4.20–11.9)**	**4.59**	**(2.80–7.55)**	**3.44**	**(2.06–5.74)**
Discrimination	Med	0.88	(0.67–1.16)	0.80	(0.48–1.33)	1.22	(0.94–1.59)	**1.37**	**(1.07–1.75)**	**2.00**	**(1.55–2.59)**
	High	3.52	(0.78–15.9)	**5.42**	**(1.02–28.6)**	1.96	(0.44–8.84)	5.61	(0.67–46.8)	4.79	(0.57–40.0)
**Model 2—partly adjusted ‡**											
Financial strain	Med	**2.78**	**(1.79–4.32)**	**4.13**	**(1.45–11.8)**	**3.34**	**(2.16–5.16)**	**2.89**	**(2.10–3.96)**	**3.58**	**(2.64–4.86)**
	High	**5.60**	**(3.40–9.24)**	**7.13**	**(2.39–21.2)**	**7.81**	**(4.77–12.8)**	**7.44**	**(4.81–11.5)**	**9.83**	**(6.21–15.6)**
Social strain	Med	**2.10**	**(1.44–3.07)**	**2.98**	**(1.31–6.77)**	**2.77**	**(1.90–4.04)**	**2.64**	**(1.96–3.56)**	**3.45**	**(2.58–4.62)**
	High	**3.54**	**(2.13–5.87)**	**5.05**	**(1.95–13.0)**	**5.13**	**(3.12–8.43)**	**8.73**	**(5.14–14.8)**	**10.9**	**(6.19–19.3)**
Competency strain	Med	**2.21**	**(1.43–3.40)**	1.49	(0.68–3.30)	**2.62**	**(1.74–3.94)**	**1.95**	**(1.44–2.64)**	**1.70**	**(1.27–2.29)**
	High	**5.10**	**(2.86–9.09)**	**3.78**	**(1.49–9.59)**	**5.04**	**(2.90–8.77)**	**3.79**	**(2.23–6.43)**	**3.65**	**(2.13–6.25)**
Discrimination	Med	1.16	(0.85–1.57)	0.98	(0.58–1.66)	**1.46**	**(1.10–1.94)**	**1.49**	**(1.15–1.94)**	**2.15**	**(1.64–2.81)**
	High	4.96	(0.81–30.3)	**6.21**	**(1.05–36.9)**	2.41	(0.46–12.6)	*	*	*	*
**Model 3—fully adjusted §**											
Financial strain	Med	**2.04**	**(1.22–3.41)**	3.40	(0.97–11.9)	**2.09**	**(1.28–3.41)**	**2.03**	**(1.41–2.94)**	**2.41**	**(1.68–3.45)**
	High	**3.14**	**(1.71–5.77)**	**5.24**	**(1.37–20.0)**	**3.97**	**(2.22–7.08)**	**3.43**	**(2.05–5.74)**	**4.53**	**(2.66–7.74)**
Social strain	Med	1.27	(0.80–2.02)	1.70	(0.65–4.43)	**1.60**	**(1.02–2.49)**	**1.72**	**(1.20–2.45)**	**2.12**	**(1.49–3.01)**
	High	1.30	(0.68–2.49)	1.72	(0.51–5.74)	1.60	(0.86–2.98)	**3.66**	**(1.96–6.83)**	**4.00**	**(2.07–7.71)**
Competency strain	Med	1.61	(0.99–2.62)	1.11	(0.45–2.70)	**1.70**	**(1.08–2.68)**	1.26	(0.89–1.79)	0.96	(0.67–1.37)
	High	**3.51**	**(1.81–6.80)**	2.40	(0.82–7.02)	**2.85**	**(1.51–5.39)**	1.64	(0.87–3.07)	1.42	(0.74–2.73)
Discrimination	Med	0.94	(0.67–1.33)	0.87	(0.47–1.61)	1.22	(0.88–1.69)	1.16	(0.86–1.57)	**1.60**	**(1.18–2.18)**
	High	2.01	(0.31–12.9)	2.77	(0.38–20.1)	0.94	(0.16–5.54)	*	*	*	*

^†^ Model 1 = univariate logistic regression (i.e., only one post-migratory stressor as independent variable in model); ^‡^ Model 2 = multivariate logistic regression, each post-migration stressor adjusted for gender, age, education, civil status and immigration year, but not the other post-migration stressors; ^§^ Model 3 = multivariate logistic regression, all four post-migration stressors and gender, age, education, civil status and immigration year in same model; * Too few participants in individual cells for parameter estimation; EQ–5D–5L answer choices dichotomized so that levels 2–5 = ‘problem’ category (code = 1) vs. level 1 = ‘no problem’ (code = 0); OR = odds ratio. Reference category = low strain. PTE = potentially traumatic experiences; bold indicates statistically significant association at the *p* < 0.05 level; Med = medium.

**Table 3:** Unadjusted and adjusted linear regression models of EQ−5L−5D index score based on Swedish time trade-off value sets.

Variable	Level	Model 1 (Unadjusted) †		Model 2 (Partly Adjusted) ‡		Model 3 (Fully Adjusted) §	
		B	95% CI	B	95% CI	B	95% CI
Financial strain	Medium	**−0.069**	**(−0.088–−0.051)**	**−0.055**	**(−0.074–−0.035)**	**−0.036**	**(−0.057–−0.016)**
	High	**−0.178**	**(−0.202–−0.155)**	**−0.154**	**(−0.180–−0.129)**	**−0.108**	**(−0.136–−0.081)**
Social strain	Medium	**−0.068**	**(−0.086–−0.050)**	**−0.057**	**(−0.076–−0.038)**	**−0.027**	**(−0.047–−0.006)**
	High	**−0.177**	**(−0.205–−0.149)**	**−0.154**	**(−0.183–−0.125)**	**−0.076**	**(−0.108–−0.044)**
Competency strain	Medium	**−0.051**	**(−0.070–−0.032)**	**−0.033**	**(−0.053–−0.013)**	−0.008	(−0.028 − 0.012)
	High	**−0.143**	**(−0.175–−0.112)**	**−0.114**	**(−0.147–−0.082)**	**−0.054**	**(−0.087–−0.021)**
Discrimination	Medium	**−0.021**	**(−0.039–−0.004)**	**−0.025**	**(−0.043–−0.008)**	−0.007	(−0.023 − 0.010)
	High	**−0.272**	**(−0.378–−0.166)**	**−0.304**	**(−0.413–−0.195)**	**−0.170**	**(−0.272–−0.068)**
Gender	Female	**−0.020**	**(−0.037–−0.002)**			**−0.032**	**(−0.049–−0.016)**
Age	30–39	−0.017	(−0.039 − 0.004)			−0.020	(−0.043 − 0.003)
	40–49	**−0.029**	**(−0.052–−0.005)**			**−0.030**	**(−0.057–−0.004)**
	≥50	**−0.103**	**(−0.128–−0.078)**			**−0.095**	**(−0.123–−0.067)**
Education	10–12 yrs	0.022	(−0.001 − 0.045)			0.011	(−0.010 − 0.032)
	13–14 yrs	0.016	(−0.008 − 0.039)			0.004	(−0.018 − 0.025)
	≥15 yrs	0.022	(−0.002 − 0.045)			0.009	(−0.012 − 0.030)
Civil status	Unmarried	0.007	(−0.011 − 0.025)			**−0.041**	**(−0.062–−0.020)**
	Divorced/widowed	**−0.065**	**(−0.106–−0.024)**			**−0.041**	**(−0.079–−0.003)**
Immigration year	2012	−0.004	(−0.042 − 0.034)			0.009	(−0.026 − 0.044)
	2013	0.006	(−0.030 − 0.042)			0.026	(−0.007 − 0.059)
PTE adversity ratio	0.20–0 29	−0.012	(−0.043 − 0.019)			0.002	(−0.026 − 0.029)
	0.30–0 39	**−0.034**	**(−0.059–−0.009)**			−0.004	(−0.027 − 0.020)
	≥0.40	**−0.066**	**(−0.088–−0.045)**			**−0.030**	**(−0.050–−0.009)**

^†^ Model 1 = univariate linear regression (i.e., models included only one predictor variable); ^‡^ Model 2 = multivariate linear regression, each post-migration stressor adjusted for gender, age, education, civil status and immigration year, but not the other post-migration stressors; ^§^ Model 3 = multivariate linear regression, all four post-migration stressors and gender, age, education, civil status and immigration year in same model; † R squared for the fully adjusted model = 0.28; PTE = potentially traumatic experiences. B = unstandardized regression coefficient. Reference categories: low (post-migration stressors), male (gender), 18–29 (age), 0–9 years (education), married (civil status), 2008–2011 (immigration year), <0.20 (PTE adversity ratio); bold indicates statistically significant association at the *p* < 0.05 level.

## Data Availability

The statistical code is available from the corresponding author. Under Swedish law and ethical approval, individual level data of this kind cannot be made publicly available.

## Supplementary Materials

The following are available online at https://www.mdpi.com/article/10.3390/ijerph19052509/s1, Figure S1: Distribution individual items post-migration stressors.
