# Supplementary material for: Post-Migration Stressors and Health-Related Quality of Life in Refugees from Syria Resettled in Sweden
Source: Int J Environ Res Public Health. 2022 Feb 22;19(5):2509. doi: 10.3390/ijerph19052509 (PMC8909133; doi:10.3390/ijerph19052509)

Figure S1. Distribution individual items post-migrations stressors
1=Never   2=Seldom   3=Sometimes   4=Often   5=Very often

3 questions comprising *financial strain*

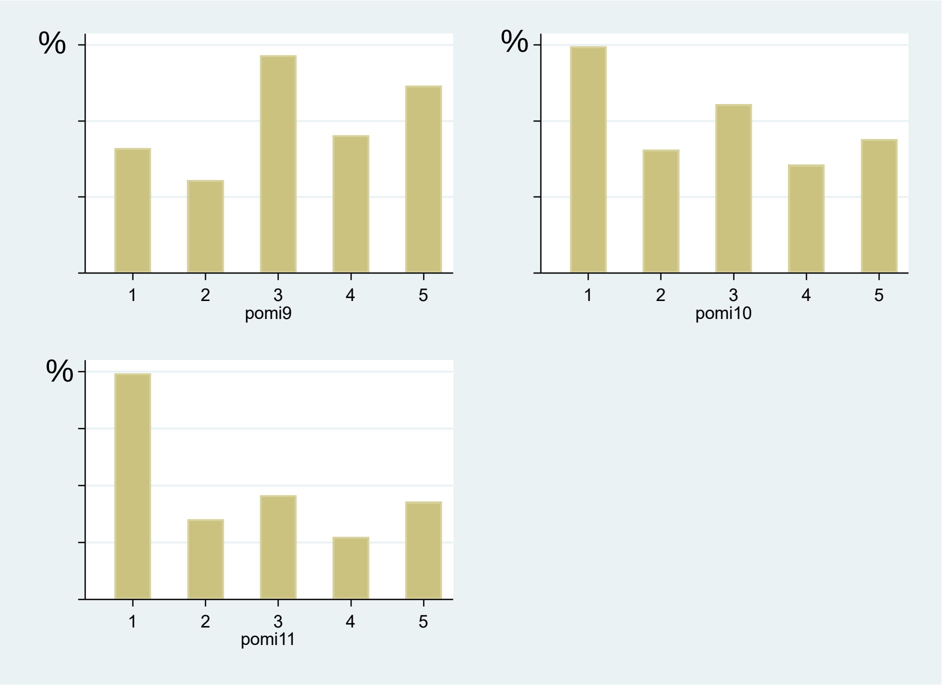

3 questions comprising *social strain*

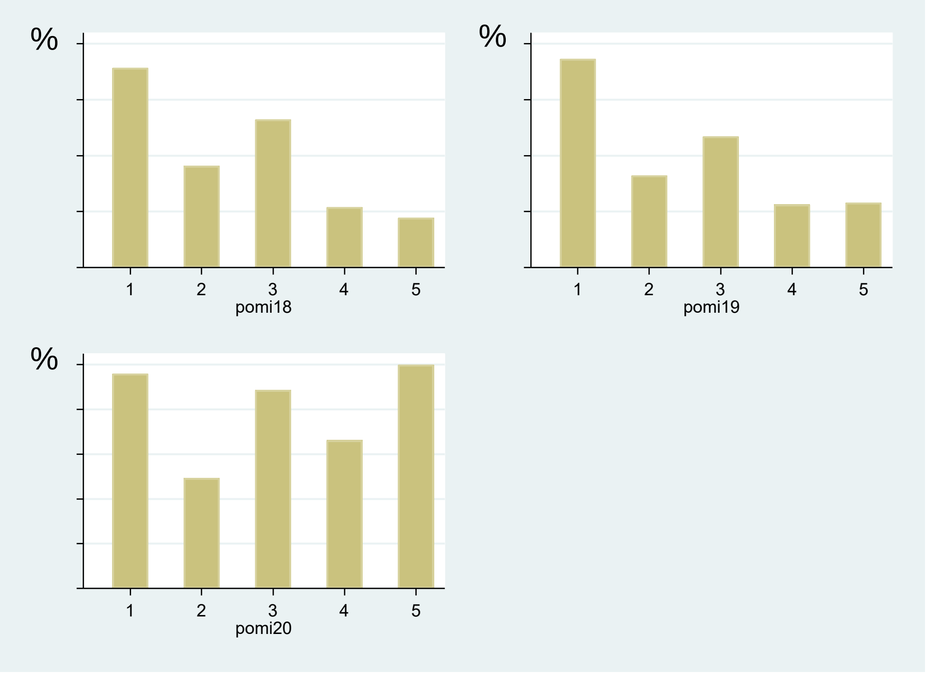

3 questions comprising *host-country competency*

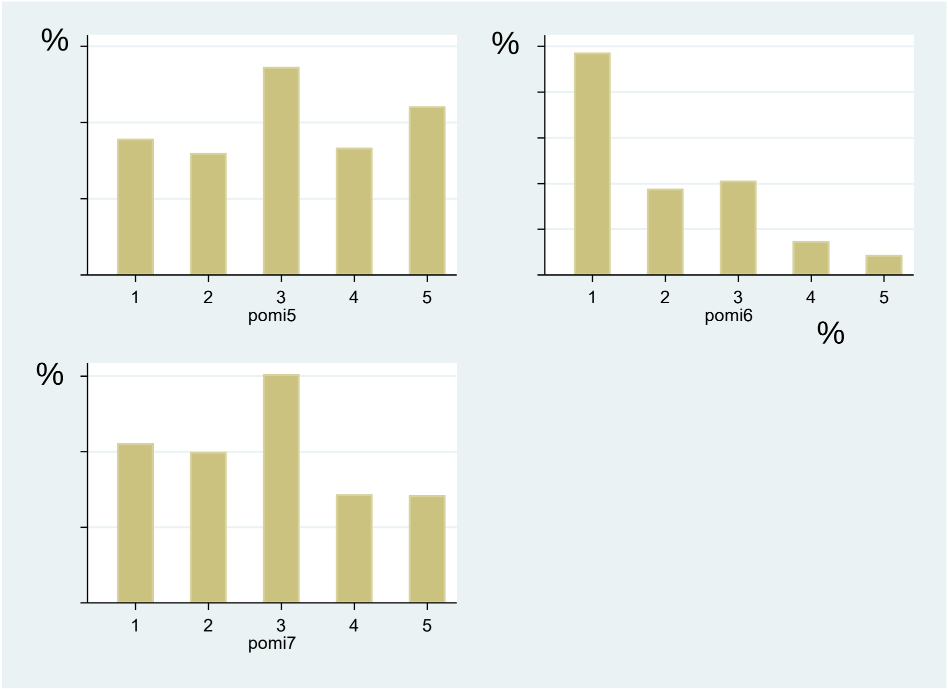

4 questions comprising *discrimination*

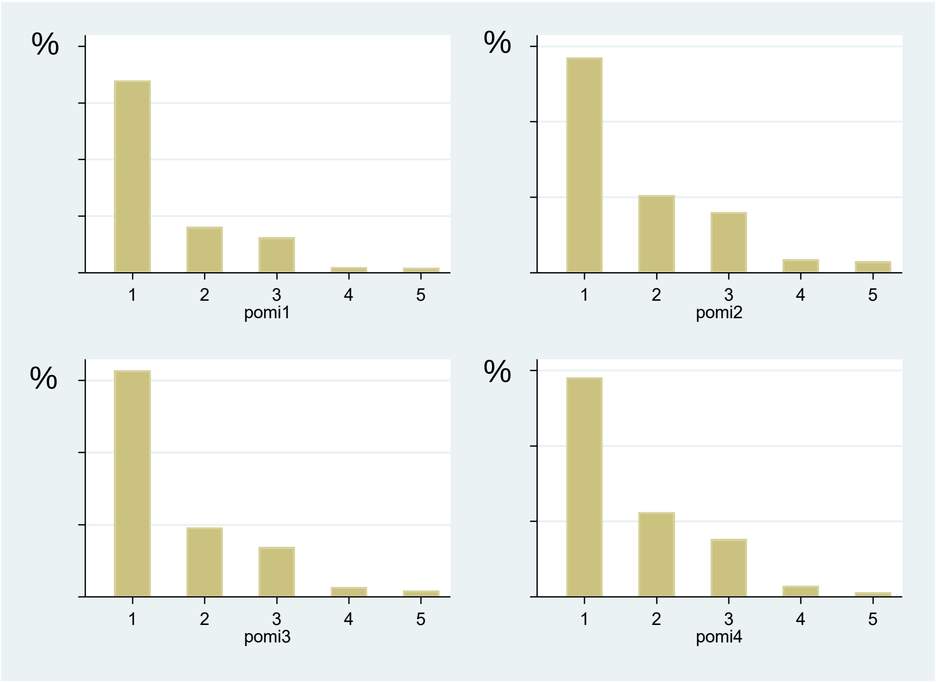

Supplement: Supplementary file 1 [file ijerph-19-02509-s001.zip › ijerph-1541414-supplementary.pdf]
